# Pre-Diagnostic Circulating Metabolites and Colorectal Cancer Risk in the Cancer Prevention Study-II Nutrition Cohort

**DOI:** 10.3390/metabo11030156

**Published:** 2021-03-09

**Authors:** Marjorie L. McCullough, Rebecca A. Hodge, Peter T. Campbell, Victoria L. Stevens, Ying Wang

**Affiliations:** Department of Population Science, American Cancer Society, Atlanta, GA 30303, USA; becky.hodge@cancer.org (R.A.H.); peter.campbell@cancer.org (P.T.C.); vstevens311@gmail.com (V.L.S.); ying.wang@cancer.org (Y.W.)

**Keywords:** metabolomics, colorectal cancer, epidemiology, biomarkers, nested case–control

## Abstract

Untargeted metabolomic studies have identified potential biomarkers of colorectal cancer risk, but evidence is still limited and broadly inconsistent. Among 39,239 Cancer Prevention Study II Nutrition cohort participants who provided a blood sample between 1998–2001, 517 newly diagnosed colorectal cancers were identified through 30 June 2015. In this nested case–control study, controls were matched 1:1 to cases on age, sex, race and date of blood draw. Mass spectroscopy-based metabolomic analyses of pre-diagnostic plasma identified 886 named metabolites, after quality control exclusions. Conditional logistic regression models estimated multivariable-adjusted odds ratios (OR) and 95% confidence intervals (CI) for 1 standard deviation (SD) increase in each metabolite with risk of colorectal cancer. Six metabolites were associated with colorectal cancer risk at a false discovery rate < 0.20. These metabolites were of several classes, including cofactors and vitamins, nucleotides, xenobiotics, lipids and amino acids. Five metabolites (guanidinoacetate, 2’-O-methylcytidine, vanillylmandelate, bilirubin (E,E) and *N*-palmitoylglycine) were positively associated (OR per 1 SD = 1.29 to 1.32), and one (3-methylxanthine) was inversely associated with CRC risk (OR = 0.79, 95% CI, 0.69–0.89). We did not replicate findings from two earlier prospective studies of 250 cases each after adjusting for multiple comparisons. Large pooled prospective analyses are warranted to confirm or refute these findings and to discover and replicate metabolites associated with colorectal cancer risk.

## 1. Introduction

Colorectal cancer (CRC) is a multi-factorial disease with many established lifestyle and behavioral risk factors, including smoking, diet, pharmacologic agents, and several variables related to energy balance and metabolism (e.g., diabetes, excess body weight, physical inactivity) [1,2,3]. Metabolomic profiling measures metabolic end products and exogenous exposures such as xenobiotics and it integrates influences of genetic variability. As such, metabolomic profiling is an ideal method to identify novel biomarkers of colorectal cancer risk. Studies have begun to identify circulating metabolomic features differentiating CRC patients from controls [4,5,6], but most collected blood after diagnosis. 

The use of metabolomic methods to identify candidate biomarkers of CRC risk is still a new area of research,[6] with few prospective studies published to date [7,8]. Identification of metabolic dysregulation biomarkers prior to CRC diagnosis may eventually lead to objective risk assessment for potential targeted prevention measures and closer screening and follow-up. Metabolites associated with CRC risk when restricting the analysis to the first 5 years of follow-up may provide clues for high risk persons and early detection of adenomas, and lead to a better understanding of the mechanisms through which risk factors play a role in CRC. The purpose of this study was to conduct a comprehensive, exploratory analysis of putative metabolomic markers of CRC risk using mass spectrometry in a nested case–control study of 517 CRC patients and 517 matched controls with pre-diagnostic blood samples from the American Cancer Society’s (ACS) Cancer Prevention Study (CPS)-II Nutrition Cohort.

## 2. Results

Participant characteristics are provided in Table 1. Due to matching, there were no differences comparing cases to controls by age, sex and race/ethnicity. Colorectal cancer cases had a significantly higher BMI and were less likely to have undergone colorectal cancer screening compared to controls. Cases also consumed more red meat and had a lower ACS dietary pattern score compared to controls. 

Associations of all 886 metabolites with CRC risk are provided in Supplementary Table S1. In Supplementary Figure S1, a heatmap illustrates interrelationships of the top 20 associated metabolites using raw p-values. Six metabolites from the multivariable-adjusted model met the criteria of false discovery rate (FDR) < 0.20 (Table 2). These included vanillylmandelate (VMA), a metabolite of epinephrine and norepinephrine and involved in tyrosine metabolism; 3-methylxanthine, a xenobiotic involved in xanthine metabolism; bilirubin (E,E), a heme breakdown product; *N*-palmitoylglycine, an acyl glycine; guanidinoacetate, an amino acid involved in creatine metabolism; and 2’-*O*-methylcytidine, a nucleotide involved in pyrimidine metabolism. The ORs for each metabolite with CRC risk were similar between minimally adjusted (including hours since last meal) and multivariable-adjusted models; therefore, only multivariable-adjusted models are presented. 3-Methylxanthine was inversely associated with risk; all other metabolites were associated with an increased CRC risk. Table 2 provides ORs and 95% CIs for each metabolite, for continuous (per SD) metabolites with and without mutual adjustment. In models simultaneously controlling for the other five metabolites, risk estimates for guanidinoacetate, vanillylmandelate, 3-methylxanthine, and 2′-*O*-methylcytidine changed least (raw *p* value < 0.05). Results using categorical variables (based on quartile distribution) are also presented. 

Only one of these six metabolites had a statistically significant interaction with sex, with a stronger association observed in men than women for 2’-*O*-methylcytidine (*p*_interaction_ = 0.04) (Table 3). When stratifying the analysis by follow-up time between blood draw and diagnosis (≤5 years, >5 years), most associations remained similar to those from the overall models but a significant interaction was noted for *N*-palmitoylglycine, with stronger associations when diagnosis occurred within the first five years of follow-up (*p*_interaction_ = 0.04)(Table 3). None of these six metabolites reached statistical significance in models stratified by tumor subsite. In analyses conducted separately by SEER stage, 2’-*O*-methylcytidine, 3-methylxanthine and guanidinoacetate were significantly associated with localized CRC tumors, and no metabolites were significantly associated with regional or distant-metastatic staged disease. However, associations were generally in the same direction regardless of tumor stage and tests of heterogeneity were all nonsignificant (Pheterogeneity ≥ 0.05, not shown).

## 3. Discussion

In this study of 517 colorectal cancer cases and 517 matched controls, six metabolites among 886 identified were moderately associated with colorectal cancer risk at the FDR < 0.20. These metabolites covered a range of metabolite classes, including amino acids, fatty acids, cofactors, nucleotides and xenobiotics. Only one metabolite association differed by sex, and sex-specific associations were generally of the same magnitude, albeit with varying precision. 

Few studies have used metabolomic approaches to identify pre-diagnostic metabolic biomarkers of CRC carcinogenesis. Of 9 studies included in a recent systematic literature review,[6] all but one analyzed biomarkers after CRC diagnosis, which can be biased by surgery, treatment, cancer progression, and changes in lifestyle after diagnosis. In these studies,[6] the number of cases ranged from 28 to 282, with 6 studies having fewer than 100 cases. Pathways that varied by case status included protein biosynthesis, urea cycle, alanine metabolism and glutathione metabolism; markers of energy and lipid metabolism also differed by case–control status.[6] Two studies that had access to pre-diagnosis blood samples (including one from the systematic review [8]) conducted untargeted metabolomic analyses using mass-spectrometry to identify biomarkers of CRC “exposotype” [7,8]. In an analysis of 254 cases and 254 matched controls in the Prostate, Lung, Colorectal and Ovarian Cancer Screening Trial cohort (PLCO), none of the 278 named metabolites measured by Metabolon’s platforms (and present in 80% of the study participants) were associated with CRC risk after adjusting for multiple comparisons, although serum glycochenodeoxycholate, a bile acid metabolite, was associated with a five-fold increased risk among women only.[8] In the current analysis, glycochenodeoxycholate was positively associated with CRC risk, but did not meet the FDR threshold (OR = 1.18, 95% CI 1.03, 1.34, *p* = 0.015, FDR = 0.39; Supplementary Table S1). The association was positive in both sexes, but slightly stronger in women (OR = 1.21, 95% CI 1.0, 1.46, *p* = 0.050, FDR = 0.61; not shown). In the Shanghai Men’s and Women’s Health Study cohorts (SMHS and SWHS) including 250 cases and 250 controls, 35 of the 618 annotated metabolites were statistically significantly associated with CRC risk using FDR < 0.05 to define significance, of which nine metabolites were independently associated with risk [7]. The strongest independent metabolite in that analysis, picolinic acid, was not replicated in the current study (picinolate, OR = 1.01, 0.88–1.15, Supplementary Table S1). In SMHS and SWHS, the metabolites most strongly associated with risk were those that play a role in glycerophospholipid dysregulation which may impact lipid profiles, energy balance, and insulin signaling.[7] We detected two lipids that were significantly inversely associated with risk in SMHS and SWHS: one (1-palmitoyl-2-docosahexaenoyl-GPC (16:0/22:6)) was not associated with risk (OR = 0.93, 95% CI 0.81, 1.06), and the other, 1-(1-enyl-palmitoyl)-2-arachidonoyl-GPE (P-16:0/20:4), a plasmalogen, was inversely associated with risk (0.86, 95% CI 0.75, 0.98, *p* = 0.024) in our study but did not meet the FDR cutpoint (FDR = 0.44). The other six metabolites were not identified in this study. Thus, two previously identified metabolites, glycochenodeoxycholate [8], a secondary bile acid formed in the large intestine by microbial flora, and 1-(1-enyl-palmitoyl)-2-arachidonoyl-GPE (P-16:0/20:4) [7], a plasmalogen, were significant at *p* < 0.05 without adjusting for multiple comparisons; these metabolites are candidates for further study.

There are several potential reasons why the studies to date have not robustly identified the same risk metabolites. These include different study populations, blood collection conditions,[9] the statistical methods employed, measurement error, limited power, and platform used. Other reasons may explain differences in statistical significance across studies. For example, in the PLCO cohort, Cross et al. [8] utilized a minimally adjusted model to identify metabolic signatures associated with CRC risk, whereas Shu et al. [7] controlled for many lifestyle variables in their models. In theory, these approaches answer a somewhat different question. Without covariates, the metabolomic signature could reflect biomarkers of multiple influences on CRC risk (genetic, lifestyle and other exposures); in the latter, controlling for known risk factors would more likely emphasize mechanisms distinct from known lifestyle risk factors, such as other environmental influences, pharmaceuticals and genetics. We present results using multivariable models to identify metabolites that are not obscured by differences in common behavioral characteristics. Nevertheless, although P-values differed, the ORs did not change in the present analysis from minimally adjusted to multivariable-adjusted models, suggesting minimal confounding from the covariables included in the models.

The metabolites identified in this study have biologically plausible roles in CRC carcinogenesis. A hallmark of cancer is impaired lipid metabolism, as was noted in the traditional case–control studies (where blood was collected after cancer diagnosis), and in the two prior nested case–control studies [7,8]. Changes in fatty acid profiles may indicate increased membrane synthesis and cellular turnover. In the current study, *N*-palmitoylglycine, a fatty acid (acyl glycine) was significantly positively associated with CRC risk, and was the only metabolite with a significantly stronger association within the first 5 years of follow-up. Whether *N*-palmitoylglycine is a risk factor involved in carcinogenesis, or a biomarker of the cancer itself is not known.

Vanillylmandelate (VMA), an organic compound used in vanilla flavor synthesis and a byproduct of epinephrine and norepinephrine metabolism, and also involved in tyrosine metabolism, was positively associated with CRC risk in this study. Mandelate, a fecal metabolite related to VMA, was associated with a three-fold increase in colorectal cancer risk in a study designed to quantify technical variability of fecal metabolomics data from 48 cases and 102 controls.[10] As altered protein synthesis has been identified as present in cancer metabolism [11], the increased risk associated with both metabolites may be relevant.

Bilirubin, a degradation product of heme which is conjugated in the liver and excreted in the bile, is a metabolic marker of liver disease, and also known to have antioxidant and potentially cytotoxic effects [12,13]. In non-metabolomic-based studies, a case–control [12] and cross-sectional study [14] reported inverse associations between blood bilirubin levels and CRC risk, whereas bilirubin was not associated with colorectal cancer risk in a German prospective cohort study (RR = 1.40, 95% CI 0.93, 2.09) [15] or a prospective analysis from NHANES [16]. In the current analysis, a bilirubin metabolite (E,E) was associated with a 29% increased risk of CRC. Tobacco use is associated with lower bilirubin levels [14]; therefore, potential confounding by tobacco should be carefully ruled out in epidemiologic analyses of bilirubin and cancer risk. Our participants were mostly non-smokers and we controlled for smoking status.

A metabolite of caffeine and theophylline, 3-methylxanthine was inversely associated with CRC risk in our study. 3-methylxanthine is a biomarker of coffee consumption [17], and coffee consumption is inversely related to CRC risk in epidemiologic analyses [18,19]. Guertin et al., observed that caffeine and theophylline both mediated the inverse association of coffee with colorectal cancer risk in the PLCO cohort [20]. Whether this metabolite plays a mechanistic role in CRC prevention, or whether it reflects other lifestyle risk factors associated with coffee consumption deserves further investigation.

Strengths of this analysis include its prospective design, large number of cases and controls, and ability to control for important CRC risk factors. Potential limitations include the one-time measurement of metabolites and processing delays in sample preparation. These factors may contribute to measurement error, which could attenuate the estimates of risk associations. However, in a previous study [21], we found good reproducibility for up to 48 h of processing delay for the five metabolites that were identified in both analyses. Some associations may also have been underestimated due to technical variation, and residual confounding due to between-person differences in fasting status, although we controlled for time since last meal to minimize the influence. Finally, with a relaxed false discovery rate *p* value cut-point, some results may be due to chance.

In summary, we identified six metabolites that were moderately associated with CRC risk in multivariable-adjusted models. These metabolites may reflect altered lipid and amino acid metabolism in carcinogenesis, and potentially other pathways including xenobiotic metabolism. Whether bilirubin and 3-methylxanthine reflect biologically meaningful mechanisms, or serve as biomarkers of exposure to CRC risk factors (e.g., red meat and coffee consumption), remains to be elucidated. To date, the limited number of prospective studies did not identify the same metabolite-CRC risk associations. Large pooled analyses of studies using similar laboratory and analytic methodology are warranted to identify and confirm candidate metabolites associated with CRC risk with greater statistical power.

## 4. Materials and Methods

### 4.1. Study Population and Design

Men and women in this study were participants in the CPS-II Nutrition Cohort, a subset of 1.2 million participants in the CPS-II Cohort (1982), who resided in 21 U.S. states when they were invited to enroll in the CPS-II Nutrition Cohort of incident cancer follow-up, beginning in 1992–1993 [22]. Between 1998–2001, 39,200 of these men and women provided a non-fasting blood sample. The Emory University School of Medicine Institutional Review Board approved all aspects of the CPS-II (Ethical Approval Code: IRB00045780).

Among those who provided blood samples, 617 CRC cases were identified through 30 June 2015 via self-report which was verified with medical records, state cancer registry linkage, or linkage with the National Death Index (defined by ICD-10 codes 18.0, 18.2–18.9, 19.9 and 20.9, excluding non-adenocarcinomas). After excluding 97 cases with prevalent cancer except for nonmelanoma skin cancer at or before blood draw, one case with an incorrect diagnosis date, and two cases with insufficient plasma, 517 cases remained [229 in men, 288 in women; 436 colon (204 proximal, 95 distal, 2 overlapping and 135 unknown), 74 rectum] and 7 unknown subsite. Controls were incidence-density matched 1:1 to cases on sex, race/ethnicity, age at blood draw (± 6 months) and date of blood draw (± 30 days).

### 4.2. Metabolomics Assessment

Metabolomic profiling was conducted by Metabolon, Inc. (Durham, NC, USA) using untargeted, ultrahigh performance liquid chromatography-tandem mass spectrometry (UPLC-MS/MS) [17,23]. Briefly, methanol was added to precipitate protein, followed by centrifugation. Four sample fractions were dried and reconstituted in different solvents for measurement under four different platforms, including two separate reversed phase (RP)/UPLC-MS/MS methods with positive ion mode electrospray ionization (ESI), one RP/UPLC-MS/MS method with negative ion mode ESI and one hydrophilic interaction chromatography (HILIC)/UPLC-MS/MS with negative ion mode ESI. Individual metabolites were identified by comparison with a chemical library maintained by Metabolon that comprises more than 3300 commercially available purified standard compounds and recurrent unknown entities, based on retention index, mass to charge ratio, and fragmentation. Peaks were quantified using area-under-the-curve and day-to-day variation corrected by setting median values for each compound to 1 for each run-day and normalizing each data point proportionately. Missing values were assumed to reflect amounts below the level of detection and were imputed to the observed minimum of the non-missing values.

Colorectal cancer cases and controls were analyzed in the same batch in a blinded fashion. Replicate quality control samples from 29 study participants were included with the study samples and used to assess intra- and inter-batch variation in the metabolite measurements. A total of 1063 named metabolites were measured. Metabolites which were undetectable in >90% of the samples (*n* = 27) and those with a technical intraclass correlation coefficient (ICC) that was missing (*n* = 74) or <0.50 (*n* = 76) were excluded from the analyses, leaving 886 named metabolites with an average CV% 0.29 (interquartile range 0.18–0.35) and ICC 0.82 (interquartile range 0.74–0.92).

### 4.3. Statistical Analysis

Metabolites were log-transformed and auto-scaled to account for non-normal distribution, consistent with our previous studies.[17] Covariates were assessed either at blood draw (1998–2001) or on the 1999 follow-up survey.

We used conditional logistic regression to estimate the odds ratios (OR) and 95% confidence intervals (CI) per one standard deviation (SD) increase of each named metabolite with CRC risk. The statistical models were conditioned on the matching factors and adjusted for: hours since last meal (to account for length of fasting), body mass index (BMI, kg/m^2^), smoking, recreational physical activity, alcohol drinking, non-steroidal anti-inflammatory drug use, American Cancer Society diet guidelines score (higher scores represent greater consumption of vegetables, whole fruit and whole grains, and lower consumption of red and processed meat intake), [24] and total energy intake (see footnote to Table 2 for details).

Associations were considered statistically significant if the false discovery rate (FDR) [25] adjusted *p* value was <0.20; this relaxed *p* value has been used in similarly sized studies [26,27] to allow for generating hypotheses. Conditional logistic regression models were used to examine associations stratified by sex and by years between blood draw and CRC diagnosis (≤5 follow-up, >5 years of follow-up). The likelihood ratio test was used to calculate *p* for interaction by comparing the full model with interaction terms to a reduced model without interaction terms. We also examined risk according to CRC tumor subsite and Surveillance, Epidemiology and End Results (SEER) stage at diagnosis: localized [invasive tumors confined to the colorectum (*n* = 195 cases)]; regional [tumors that extend through the bowel wall to adjacent tissue or regional lymph nodes (*n* = 225 cases)] and distant metastases (*n* = 53). The Wald *p* value for heterogeneity by tumor site and stage was estimated from an unconditional nominal polytomous logistic regression model using the model-based variance–covariance matrix estimate [28]. Analyses were conducted using R version 4.0.2 (The R Foundation for Statistical Computing, Vienna, Austria) [29], and SAS version 9.4 (SAS Institute, Cary, NC, USA).

## Figures and Tables

**Table 1:** Participant characteristics of a nested, matched ^a^ case–control study in the Cancer Prevention Study-II Nutrition Cohort.

	Cases (*n* = 517)	Controls (*n* = 517)	*p*-Value ^b^
Age at Blood Draw ^c^, Mean (SD)	70.2 (5.5)	70.2 (5.5)	Matched
Sex, *n* (%)			Matched
Male	229 (44.3)	229 (44.3)	
Female	288 (55.7)	288 (55.7)	
Race, *n* (%)			Matched
White	505 (97.7)	506 (97.9)	
Black	4 (0.8)	4 (0.8)	
Other/Unknown	8 (1.5)	7 (1.4)	
Highest Education Level, *n* (%)			0.217
Less than High School	13 (2.5)	12 (2.3)	
High School Grad	130 (25.1)	107 (20.7)	
Some College	157 (30.4)	169 (32.7)	
College Grad	113 (21.9)	111 (21.5)	
Grad School	101 (19.5)	118 (22.8)	
Unknown	3 (0.6)	0 (0.0)	
Body Mass Index (kg/m^2^), Mean (SD)	26.5 (4.7)	25.7 (4.1)	0.004
Hours Since Last Meal ^c^, Mean (SD)	2.3 (2.2)	2.2 (1.9)	0.232
Smoking Status, *n* (%)			0.773
Never	245 (47.4)	251 (48.5)	
Former	247 (47.8)	247 (47.8)	
Current	14 (2.7)	12 (2.3)	
Unknown	11 (2.1)	7 (1.4)	
Physical Activity, (MET-h/week), *n* (%)			0.825
<8.75	194 (37.5)	189 (36.6)	
8.75–<17	143 (27.7)	137 (26.5)	
17+	173 (33.5)	181 (35.0)	
Missing	7 (1.4)	10 (1.9)	
Alcohol Consumption ^c^, *n* (%)			0.662
<1 Drink/D	408 (78.9)	410 (79.3)	
1+ Drinks/D	97 (18.8)	99 (19.1)	
Unknown	12 (2.3)	8 (1.5)	
NSAID User			0.161
No	213 (41.2)	190 (36.8)	
Yes	304 (58.8)	327 (63.2)	
Postmenopausal Hormone Use ^c,d^, n (%)			0.093
Not a Current User	168 (58.3)	142 (49.3)	
Current User	118 (41.0)	144 (50.0)	
Unknown	2 (0.1)	2 (0.1)	
Cancer Subsite			N/A
Control	0 (0.0)	515 (99.6)	
Proximal Colon	204 (39.5)	2 (0.4)	
Distal Colon	95 (18.4)	0 (0.0)	
Rectum	74 (14.3)	0 (0.0)	
Colon	137 (26.5)	0 (0.0)	
Unknown	7 (1.4)	0 (0.0)	
Cancer stage			N/A
Control	0 (0.0)	515 (99.6)	
Local	195 (37.7)	1 (0.2)	
Regional	225 (43.5)	1 (0.2)	
Distant (Metastatic)	53 (10.3)	0 (0.0)	
Unknown	44 (8.5)	0 (0.0)	
Colorectal Screening			0.003
Never Screened	159 (30.8)	120 (23.2)	
Screened in the Past	302 (58.4)	355 (68.7)	
Unknown Screening Status	56 (10.8)	42 (8.1)	
Diet and Nutrients, Mean (SD)			
Red Meat (servings/day)	0.7 (0.5)	0.6 (0.4)	0.042
Processed Meat (servings/day)	0.3 (0.3)	0.3 (0.3)	0.769
Caffeinated Coffee (drinks/day)	0.9 (1.2)	0.9 (1.3)	0.961
Decaffeinated Coffee (drinks/d)	0.6 (0.9)	0.6 (1.0)	0.770
Total Folate (mcg)	620 (287)	625 (282)	0.788
Dietary Fiber (g)	18.7 (7.0)	18.8 (7.2)	0.734
Total calcium (mg)	1102 (555)	1161 (537)	0.091
Total Vitamin D (IU)	395 (246)	418 (246)	0.138
Total Calories	1725 (493)	1734 (549)	0.769
Diet Score, mean (SD)			
ACS Diet Score (range: 0–9 patients)	4.3 (1.9)	4.6 (2.0)	0.039

^a^ Controls were matched to cases according to sex, race, age and date of blood draw. Two matched controls later became cases. ^b^ *p*-values obtained from chi squared tests (categorical) or independent t-tests (continuous). ^c^ These variables were obtained from the Lifelink biospecimen collection survey (1997 and 1998); sex, race and education were from the 1982 CPS-II baseline survey; all others were from the CPS-II Nutrition Cohort 1999 follow-up survey. ^d^ Numbers are for women only.

**Table 2:** OR and 95% CI for metabolites associated with colorectal cancer risk at FDR < 0.2 in the Cancer Prevention Study II Nutrition Cohort (*n* = 517 matched cases and controls).

	Guanidinoacetate	Vanillylmandelate (VMA)	3-methylxanthine	2’-*O*-methylcytidine	Bilirubin (E,E)	*N*-palmitoylglycine
Multivariable Adjusted Continuous Model ^a^						
Per SD	1.32 (1.14, 1.52)	1.29 (1.12, 1.49)	0.79 (0.69, 0.89)	1.27 (1.11, 1.46)	1.29 (1.11, 1.50)	1.27 (1.11, 1.45)
*p*	<0.001	<0.001	<0.001	<0.001	0.001	0.001
FDR	0.090	0.090	0.090	0.090	0.121	0.106
Continuous, Mutually Adjusted Model ^b^						
Per SD	1.24 (1.07, 1.45)	1.28 (1.09, 1.49)	0.74 (0.64, 0.85)	1.18 (1.02, 1.36)	1.15 (0.97, 1.36)	1.12 (0.96, 1.30)
*p*	0.005	0.002	<0.001	0.028	0.099	0.139
Multivariable Adjusted Quartiles ^a^						
Q1 (ref)	1.00 (ref)	1.00 (ref)	1.00 (ref)	1.00 (ref)	1.00 (ref)	1.00 (ref)
Q2	1.26 (0.86, 1.83)	1.64 (1.13, 2.40)	0.63 (0.44, 0.91)	1.26 (0.89, 1.80)	1.10 (0.76, 1.60)	1.23 (0.85, 1.78)
Q3	1.42 (0.98, 2.06)	1.51 (1.04, 2.18)	0.65 (0.46, 0.93)	1.71 (1.18, 2.47)	1.51 (1.02, 2.24)	1.40 (0.97, 2.01)
Q4	1.87 (1.26, 2.77)	1.94 (1.31, 2.89)	0.51 (0.35, 0.73)	1.73 (1.20, 2.50)	1.64 (1.10, 2.45)	1.97 (1.36, 2.87)
*p*_trend_^c^	0.001	0.003	<0.001	0.001	0.008	<0.001
FDR	0.220	0.236	0.220	0.220	0.272	0.220

Note: CI, confidence interval; FDR, false discovery rate-adjusted *p* values; OR, odds ratio; SD, standard deviation. ^a^ Odds ratios (95% confidence intervals) were estimated from conditional logistic regression models, matched on sex, race, age, and date of blood draw. Models were adjusted for hours since last meal (continuous), body mass index at blood draw (continuous, kg/m^2^), smoking status in 1999 (never, former, current, unknown), recreational physical activity in 1999 in metabolic equivalent (MET)-h/week (<8.75; 8.75–<17; 17+, unknown), alcohol consumption in 1999 (nondrinker, <1 drink/day, 1+ drinks/day, unknown), current NSAID use in 1999 (yes, no), ACS diet score (tertiles, comprised of scores for red and processed meat, proportion of whole vs. refined grains consumed and fruit and vegetable consumption plus a missing category), and total calories (continuous). Individuals with missing continuous variables (BMI, calories) were assigned the study median value. ^b^ Multivariable model, additionally adjusted for the other five metabolites. ^c^ *p* for trend based on median values in each quartile.

**Table 3:** OR and 95% CI for metabolites associated with colorectal cancer risk at FDR < 0.2 in the Cancer Prevention Study II Nutrition Cohort, stratified by follow-up time and participant sex^a.^

Model	Guanidinoacetate	Vanillylmandelate (VMA)	3-methylxanthine	2’-*O*-methylcytidine	Bilirubin (E,E)	*N*-palmitoylglycine
*Individual Metabolites (continuous)*						
Follow-Up ≤5 years (229 cases)						
Per SD	1.22 (0.99, 1.52)	1.34 (1.08, 1.66)	0.79 (0.65, 0.97)	1.30 (1.06, 1.60)	1.21 (0.97, 1.51)	1.47 (1.18, 1.83)
*p*	0.067	0.008	0.024	0.011	0.095	0.001
Follow-Up >5 years (288 cases)						
Per SD	1.42 (1.17, 1.74)	1.26 (1.03, 1.53)	0.76 (0.63, 0.90)	1.27 (1.05, 1.53)	1.35 (1.09, 1.66)	1.15 (0.96, 1.37)
*p*	0.001	0.023	0.002	0.014	0.006	0.138
*p*_interaction_	0.582	0.591	0.717	0.537	0.550	0.042
Men Only (229 cases)						
Per SD	1.52 (1.21, 1.91)	1.33 (1.08, 1.64)	0.84 (0.68, 1.03)	1.54 (1.23, 1.92)	1.32 (1.05, 1.66)	1.19 (0.96, 1.47)
*p*	<0.001	0.007	0.093	<0.001	0.018	0.109
Women Only (288 cases)						
Per SD	1.20 (0.99, 1.44)	1.25 (1.02, 1.53)	0.74 (0.63, 0.88)	1.11 (0.93, 1.33)	1.29 (1.05, 1.58)	1.34 (1.12, 1.61)
*p*	0.063	0.028	0.001	0.252	0.015	0.002
*p*_interaction_	0.173	0.741	0.341	0.043	0.849	0.343

Note: CI, confidence interval; FDR, false discovery rate-adjusted *p* values; OR, odds ratio; SD = standard deviation. *^a^* Odds ratios (95% confidence intervals) estimated from conditional logistic regression models, matched on sex (except sex-stratified models), race, age, and date of blood draw. Models controlled for hours since last meal (continuous), body mass index at blood draw (continuous), smoking status in 1999 (never, former, current, unknown), recreational physical activity in 1999 in metabolic equivalent (MET)-h/week (<8.75, 8.75–<17, 17+, unknown), alcohol consumption in 1999 (nondrinker, <1 drink/day, 1+ drinks/day, unknown), current NSAID use in 1999 (yes, no), ACS diet score (tertiles, comprised of scores for red and processed meat, proportion of whole vs. refined grains consumed and fruit and vegetable consumption plus a missing category) and total calories (continuous). Individuals with missing continuous variables (BMI, calories) were assigned the study median value.

## Data Availability

Data described in the manuscript and analytic code are not available to protect participant confidentiality and in adherence with institutional policies.

## Supplementary Materials

The following are available online at https://www.mdpi.com/2218-1989/11/3/156/s1, Table S1: Individual metabolite (*n* = 886) associations with colorectal cancer risk in the CPS-II Nutrition Cohort (*n* = 517 matched cases and controls), Figure S1: Interrelationships of top 20 CRC-associated metabolites priorbased on raw *p*-values.

## References

[1] Campbell P.T. (2013). The role of diabetes and diabetes treatments in colorectal cancer mortality, incidence and survival. Curr. Nutr. Rep..

[2] Murphy N., Jenab M., Gunter M.J. (2018). Adiposity and gastrointestinal cancers: Epidemiology, mechanisms and future directions. Nat. Rev. Gastroenterol. Hepatol..

[3] Wu K., Keum N., Nishihara R., Giovannucci E.L., Thun M.J., Linet M.S., Cerhan J.R., Haiman C.A., Schottenfeld D. (2018). Cancers of the Colon and Rectum. Cancer Epidemiology and Prevention.

[4] Farshidfar F., Weljie A.M., Kopciuk A.K., Hilsden R., McGregor S.E., Buie W.D., MacLean A., Vogel H.J., Bathe O.F. (2016). A validated metabolomic signature for colorectal cancer: Exploration of the clinical value of metabolomics. Br. J. Cancer.

[5] Zhang F., Zhang Y., Zhao W., Deng K., Wang Z., Yang C., Ma L., Openkova M.S., Hou Y., Li K. (2017). Metabolomics for biomarker discovery in the diagnosis, prognosis, survival and recurrence of colorectal cancer: A systematic review. Oncotarget.

[6] Hashim N.A.A., Ab-Rahim S., Suddin L.S., Saman M.S.A., Mazlan M. (2019). Global serum metabolomics profiling of colorectal cancer. Mol. Clin. Oncol..

[7] Shu X., Xiang Y.-B., Rothman N., Yu D., Li H.-L., Yang G., Cai H., Ma X., Lan Q., Gao Y.-T. (2018). Prospective study of blood metabolites associated with colorectal cancer risk. Int. J. Cancer.

[8] Cross A.J., Moore S.C., Boca S., Huang W.-Y., Xiong X., Stolzenberg-Solomon R., Sinha R., Sampson J.N. (2014). A prospective study of serum metabolites and colorectal cancer risk. Cancer.

[9] Sampson J.N., Boca S.M., Shu X.O., Stolzenberg-Solomon R.Z., Matthews C.E., Hsing A.W., Tan Y.T., Ji B.-T., Chow W.-H., Cai Q. (2013). Metabolomics in epidemiology: Sources of variability in metabolite measurements and implications. Cancer Epidemiol. Biomark. Prev..

[10] Goedert J.J., Sampson J.N., Moore S.C., Xiao Q., Xiong X., Hayes R.B., Ahn J., Shi J., Sinha R. (2014). Fecal metabolomics: Assay performance and association with colorectal cancer. Carcinogenesis.

[11] Lieu E.L., Nguyen T., Rhyne S., Kim J. (2020). Amino acids in cancer. Exp. Mol. Med..

[12] Jirásková A., Novotný J., Novotný L., Vodička P., Pardini B., Naccarati A., Schwertner H.A., Hubáček J.A., Punčochářová L., Šmerhovský Z. (2012). Association of serum bilirubin and promoter variations in HMOX1 and UGT1A1 genes with sporadic colorectal cancer. Int. J. Cancer.

[13] Peng Y.-F., Goyal H., Xu G.-D. (2017). Serum bilirubin has an important role in multiple clinical applications. J. Lab. Precis. Med..

[14] Zucker S.D., Horn P.S., Sherman K.E. (2004). Serum bilirubin levels in the U.S. population: Gender effect and inverse correlation with colorecal cancer. Hepatology.

[15] Kuhn T., Sookthai D., Graf M.E., Schubel R., Freisling H., Johnson T., Katzke V., Kaaks R. (2017). Albumin, bilirubin, uric acid and cancer risk: Results from a prospective population-based study. Br. J. Cancer.

[16] Ioannou G.N., Liou I.W., Weiss N.S. (2006). Serum bilirubin and colorectal cancer risk: A population-based cohort study. Aliment. Pharmacol. Ther..

[17] Wang Y., Gapstur S.M., Carter B.D., Hartman T.J., Stevens V.L., Gaudet M.M., McCullough M.L. (2018). Untargeted metabolomics identifies novel potential biomarkers of habitual food intake in a cross-sectional study of postmenopausal women. J. Nutr..

[18] Gapstur S.M., Anderson R.L., Campbell P.T., Jacobs E.J., Hartman T.J., Hildebrand J.S., Wang Y., McCullough M.L. (2017). Associations of Coffee Drinking and Cancer Mortality in the Cancer Prevention Study-II. Cancer Epidemiol. Biomark. Prev..

[19] Um C.Y., McCullough M.L., Guinter M.A., Campbell P.T., Jacobs E.J., Gapstur S.M. (2020). Coffee consumption and risk of colorectal cancer in the Cancer Prevention Study-II Nutrition Cohort. Cancer Epidemiol..

[20] Guertin K.A., Loftfield E., Boca S.M., Sampson J.N., Moore S.C., Xiao Q., Huang W.-Y., Xiong X., Freedman N.D., Cross A.J. (2015). Serum biomarkers of habitual coffee consumption may provide insight into the mechanism underlying the association between coffee consumption and colorectal cancer. Am. J. Clin. Nutr..

[21] Wang Y., Carter B.D., Gapstur S.M., McCullough M.L., Gaudet M.M., Stevens V.L. (2018). Reproducibility of non-fasting plasma metabolomics measurements across processing delays. Metabolomics.

[22] Calle E.E., Rodriguez C., Jacobs E.J., Almon M.L., Chao A., McCullough M.L., Feigelson H.S., Thun M.J. (2002). The American Cancer Society Cancer Prevention Study II Nutrition Cohort—Rationale, Study Design, and Baseline Characteristics. Cancer.

[23] Evans A.M., DeHaven C.D., Barrett T., Mitchell M., Milgram E. (2009). Integrated, nontargeted ultrahigh performance liquid chromatography/electrospray ionization tandem mass spectrometry platform for the identification and relative quantification of the small-molecule complement of biological systems. Anal. Chem..

[24] McCullough M.L., Patel A.V., Kushi L.H., Patel R., Willett W.C., Doyle C., Thun M.J., Gapstur S.M. (2011). Following cancer prevention guidelines reduces risk of cancer, cardiovascular disease, and all-cause mortality. Cancer Epidemiol. Biomark. Prev..

[25] Benjamini Y., Hochberg Y. (1995). Controlling the false discovery rate: A practical and powerful approach to multiple testing. J. R. Stat. Soc. Ser. B Methodol..

[26] Moore S.C., Playdon M.C., Sampson J.N., Hoover R.N., Trabert B., Matthews E.C., Ziegler R.G. (2018). A Metabolomics Analysis of Body Mass Index and Postmenopausal Breast Cancer Risk. J. Nat. Cancer Inst..

[27] Wang Y., Jacobs E.J., Carter B.D., Gapstur S.M., Stevens V.L. (2019). Plasma Metabolomic Profiles and Risk of Advanced and Fatal Prostate Cancer. Eur. Urol. Oncol..

[28] Wang M., Spiegelman D., Kuchiba A., Lochhead P., Kim S., Chan A.T., Poole E.M., Tamimi R.M., Tworoger S.S., Giovannucci E. (2016). Statistical methods for studying disease subtype heterogeneity. Stat. Med..

[29] R Core Team R: A Language and Environment for Statistical Computing. https://www.R-project.org/.

