# Supplementary figures and images for: Pre-Diagnostic Circulating Metabolites and Colorectal Cancer Risk in the Cancer Prevention Study-II Nutrition Cohort
Source: Metabolites. 2021 Mar 9;11(3):156. doi: 10.3390/metabo11030156 (PMC8000483; doi:10.3390/metabo11030156)

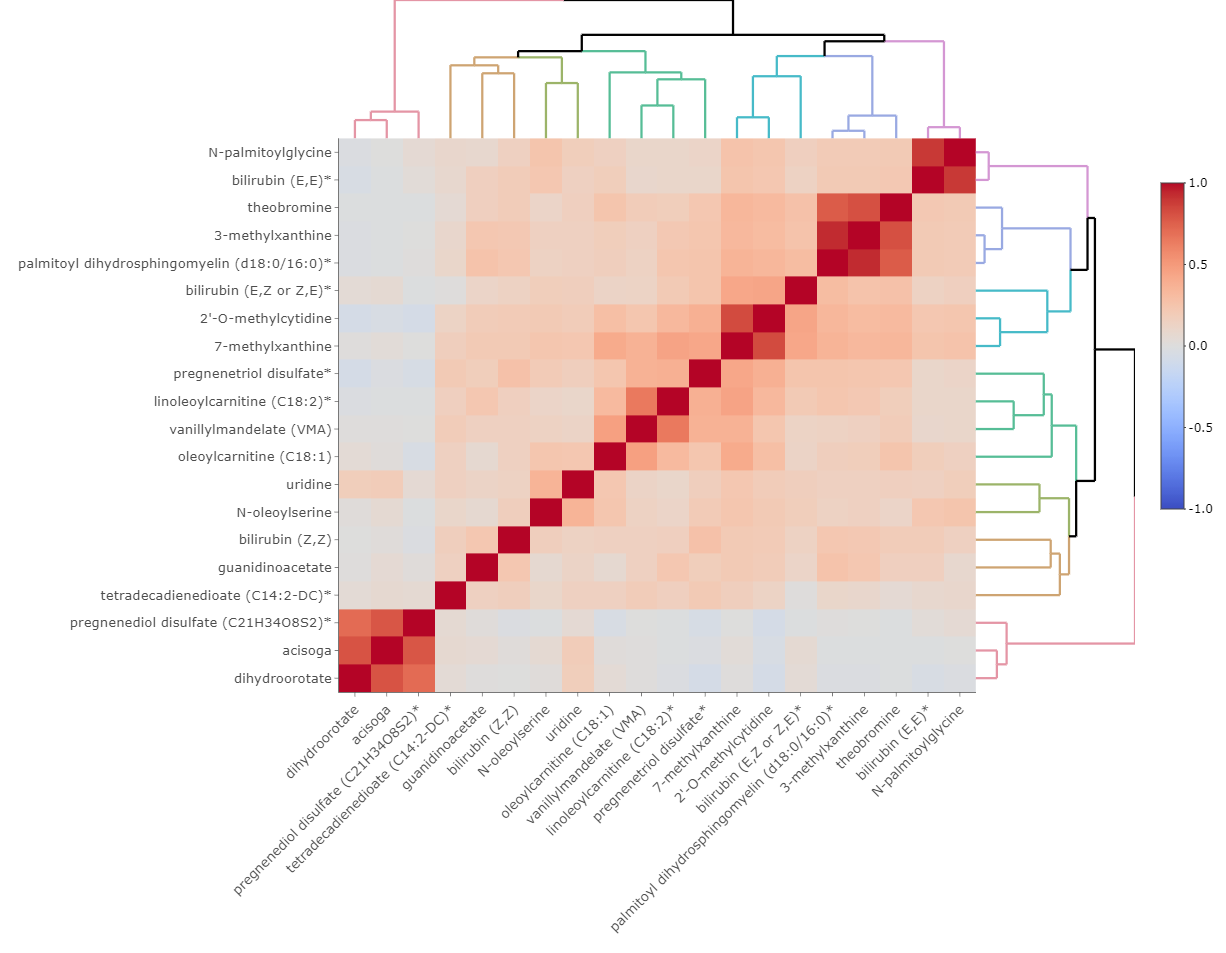


**Figure S1.** Interrelationships of top 20 CRC-associated metabolites prior to FDR adjustment.

Supplement: Supplementary file 1 [file metabolites-11-00156-s001.zip › metabolites-1126360 - supplemental figure S1.docx]
